# The evaluation of stomatology English education in China based on ‘Guanghua cup’ international clinical skill exhibition activity

**DOI:** 10.1186/s12909-020-02389-7

**Published:** 2020-11-26

**Authors:** Xi Wang, Yangjingwen Liu, Le Yang, Kai Zhou, Yang Cao, Yun Hong, Shuheng Huang, Zhengmei Lin

**Affiliations:** Hospital of Stomatology, Guanghua School of Stomatology, Guangdong Provincial Key Laboratory of Stomatology, Sun Yat-Sen University, 56 Lingyuan Road West, Guangzhou, 510055 Guangdong China

**Keywords:** Stomatology English education, Guanghua cup activity, Chinese stomatology education, Clinical skill competition

## Abstract

**Background:**

English education in professional areas has become more and more important with the increasing internationalization of health profession education in countries around the world. In this study, we aimed to evaluate current Chinese stomatology English education based on Chinese participants’ ability to apply stomatology English during an international stomatology skill competition called the ‘Guanghua Cup activity’.

**Methods:**

The registration rate of English and Chinese volunteers and the answer rate and accuracy of Chinese and international contestants on the English knowledge quiz were statistically described. A five-point Likert scale questionnaire was delivered to all participants. The data were analyzed using the Spearman test, Kruskal-Wallis rank sum test and Mann-Whitney U test.

**Results:**

Among the 194 students, the English and Chinese volunteer registration rate was 7.73 and 30.93%, respectively. The answer rate of Chinese contestants and international contestants in the English quiz was 25 and 75%, with an accuracy rate of 50 and 66.70%, respectively. The questionnaire was graded by Likert five-level classification. There was a positive correlation between the use of English textbooks in classes and the communication with international teachers and students in the competition (Rs = 0.348, *p* = 0.016). English volunteers had more preparation in English before the competition, more opportunities to communicate with international peers, and greater improvement in English ability than the contestants and Chinese volunteers (*p* < 0.001). After the competition, all participants paid more attention to stomatology English (*p* < 0.001).

**Conclusions:**

Chinese stomatology students have difficulty in stomatology English application. The ‘Guanghua Cup’ helps to improve English proficiency of English volunteers and arouses the interest of stomatology English for all participants. Chinese stomatology school needs to strengthen and reach a consensus in stomatology English education.

## Background

As the official language of international conferences, academic journals and websites, English brings challenges to students and health professionals from non-English-speaking countries [1, 2]. With the increasing internationalization of health profession education in countries around the world, English education in professional areas has become increasingly important [3]. Studies have shown that in non-English-speaking countries such as Japan, competency and confidence in the English language could be one of the largest obstacles for stomatology students to study abroad [4]. Poor English ability may limit students’ horizons and possibilities in careers for stomatology students [5]. Therefore, the demand for stomatology English education to allow students to communicate with the international peers and pursue further career goals has increased. Moreover, as the number of visas and permanent residence permits issued to foreigners increased steadily every year, [6] Chinese professionals were facing language barrier in providing quality stomatology care to foreign patients. However, whether the current stomatology English education can meet the demand for English application of Chinese stomatology students was unknown.

In China, college English education can be divided into two stages. Stage one was general English education with standardized national English examinations in universities across China that required university students to achieve a certain level in general English, including listening, reading and writing ability [7]. Stage two was English education in professional areas, which was led by universities. In the field of stomatology in China, most stomatology English courses were conducted together with other stomatology courses after the third year of stomatology school. The stomatology English course was established on the basis of college general English education. This course often involves technical topics related to stomatology in order to better prepare stomatology students to understand the literature and to communicate at international dental conferences or with foreign patients in clinical settings. According to a survey on stomatology English education in the six top stomatology schools of China, a stomatology English course was implemented in 1981 at Sichuan University, while the other five universities implemented this course in approximately 1995. Textbooks, teaching methods, class hours and teaching staff vary among universities [8]. Other stomatology schools did not have stomatology English courses until recent years because of a lack of attention and resouces [9]. Based on the limited studies, stomatology schools in China differed in their resources and teaching strategies for stomatology English education [8–10]. An overall description of stomatology English education and an evaluation of the English ability of stomatology students in China was lacking.

From 2017 to 2019, Guanghua School of Stomatology, Sun Yat-sen University held a stomatology clinical skill competition called the ‘Guanghua Cup’ clinical skills exhibition activity for 3 years (hereinafter referred to as the ‘Guanghua Cup activity’). In 2017 and 2018, more than 20 stomatology schools across the country were invited. In 2019, stomatology schools from all around China as well as schools from the United States and Thailand were involved. Therefore, all aspects of the Guanghua Cup activity in 2019 required the participants to have proficiency in stomatology English. This activity provides a platform for participants to put stomatology English into actual practice. The purpose of this study is to explore the effects of international competitions such as the Guanghua Cup activity on the stomatology English ability of the participants and to evaluate and analyze existing stomatology English education in China.

## Methods

### Ethical approval and consent to participate

Ethical approval was obtained from the Medical Ethics Committee of the Hospital of Stomatology, Sun Yat-sen University (Institutional Review Board no. KQEC-2020-11). The committee waived the need for written informed consent because responding to this anonymized questionnaire involved no more than minimal risk, and consent by action was adopted.

### Basic information about the Guanghua cup activity

The Guanghua Cup activity was held in mid-December 2019. A total of 22 teams (4 contestants in each team) from the top stomatology schools in the Beijing, Shanghai, Jilin, Sichuan, Shanxi, Xinjiang, Zhejiang, Nanjing, Heilongjiang, Liaoning, Hubei, Chongqing, Guangdong and Guangxi provinces of China as well as two international teams from Thailand and the United States participated in the event. 

Before the event, the contestants were recruited among 90 students in grade-five undergraduates, and English and Chinese volunteers were recruited among 194 grade-five undergraduates and postgraduates from the Guanghua School of Stomatology, Sun Yat-sen University, with no limits on the number or candidate qualifications. English volunteers included English hosts who were responsible for hosting the event in English, English receptionists who were responsible for contacting wiith the teams from Thailand and the United States, and translators who were responsible for simultaneous translation during the event. Chinese volunteers included Chinese hosts who were responsible for hosting the event in Chinese, Chinese receptionists who were responsible for contacting with the teams from China, and invigilators who were responsible for maintaining order during the event. Finally, 11 English volunteers, 58 Chinese volunteers and four contestants from Guanghua School of Stomatology, Sun Yat-sen University were chosen based on their willingness and the teachers’ assessment.

The Guanghua Cup activity had two sessions: a clinical operation skill assessment session and a knowledge quiz session. The questions in the knowledge quiz session were developed by the 24 teams; each team provided four questions before the event. Since there were two international teams, a total of eight questions were in English. The rest of the questions provided by the Chinese teams were bilingual. Because the teams providing the questions were forbidden to answer their own questions, 22 Chinese teams and one of the two international teams vied to answer the eight questions in English by using the buzzers.

### Questionnaire survey

The questionnaire used in the study was developed for this study and has not previously been published elsewhere. It was conducted after the event. The questionnaires were delivered to all the participants via WeChat. The questionnaire included 16 five-point Likert scale items, one single-choice item and one multiple-choice item with open answers. The options ranged from strongly disagree (one point) to strongly agree (five points) or five other classification descriptions, calculated on a scale of one to five. Three main themes were assessed using the questionnaire: 1) the current situation of stomatology English education in China; 2) the influence of stomatology English education on the activities; and 3) the influence of the Guanghua Cup activity on the perceptive improvement of the participants’ English ability. The questionnaire was collected, and unqualified questionnaires were eliminated.

### Statistical analysis

SPSS 19.0 statistical software was used to analyze the data. Descriptive statistics were applied to present the registration rate of Chinese and English volunteers, the answer rate and accuracy of English questions answered by Chinese and international contestants and the questionnaire survey. Internal consistency through a pilot study was analyzed using the Cronbach’s alpha coefficient. The internal consistency assessment yielded an overall Cronbach’s alpha coefficient of 0.78, indicating that the questionnaire generally showed internal consistency.

The Likert questionnaire was calculated on a scale of one to five. The stomatology English education scores listed in Table 4 were defined by the author as the average scores of the amount of English used in English stomatology courses for each stomatology school listed in Table 3. A total of 2-4 contestants from each school completed the questionnaire. Descriptive statistics were summarized as frequencies and percentages. The Spearman rank correlation test was used to determine the correlation of current stomatology English education and the perception of the participants’ stomatology English ability as well as the correlation of the stomatology English education score of each stomatology school and the ranking of the stomatology school. The Kruskal-Wallis rank sum test was used to compare English and Chinese volunteers and contestants in terms of English ability and perceptual English ability improvement. The Mann-Whitney U test was used to compare the English ability of undergraduate and postgraduate students as well as stomatology English perception before and after the Guanghua Cup activity. A *P*-value of 0.05 was considered statistically significant. However, we consider this study quite exploratory given the limited number of participants in the Guanghua cup activity. Hence, we have commented upon trends in the data when comparative results were within the borderline of statistical significance.

## Results

### Volunteer registration

A total of 194 senior undergraduates and postgraduates (age: 22.23 ± 1.94 years; female: 130, male: 64) from the Guanghua School of Stomatology, Sun Yat-sen University were volunteer candidates. There were 15 students who registered for the English volunteer, accounting for 20% of the total number of registration students and 7.73% of the total number of volunteer candidates. There were 60 students who registered for the Chinese volunteer, accounting for 80% of the registration students and 30.93% of the total number of volunteer candidates.

### Sample characteristics of the questionnaire survey

A total of 121 questionnaires were released and 116 valid questionnaires were collected, with a response rate of 95.87%. Since the students were not required to answer all the questions, unanswered questions were considered missing data.

The characteristics of the 116 participants (age 22 ± 1.7) from 22 top stomatology schools in China are summarized in Table 1.

**Table 1:** Sample characteristics of participants (*n* = 116)

Sample characteristics	Number(%)
Participants	
Contestants	80 (68.97)
English volunteers	9 (7.76)
Chinese volunteers	27 (23.27)
Gender	
Male	28 (24.14)
Female	88 (75.86)
University of stomatology school	
Sun Yat-sen University	36 (31.03)
Capital Medical University	4 (3.44)
Zhejiang University	3 (2.56)
Shanghai Jiao Tong University	3 (2.56)
Nanjing Medical University	4 (3.44)
Guangxi Medical University	4 (3.44)
Shandong University	3 (2.56)
Harbin Medical University	4 (3.44)
Air Force Medical University	4 (3.44)
Wuhan University	4 (3.44)
Tianjin Medical University	4 (3.44)
Peking University	4 (3.44)
Jilin University	4 (3.44)
Guangzhou Medical University	4 (3.44)
Southern Medical University	5 (4.31)
Dalian Medical University	4 (3.44)
Chongqing Medical University	4 (3.44)
Sichuan university	2 (1.72)
Xian Jiaotong University	4 (3.44)
Tongji University	4 (3.44)
Xinjiang Medical University	4 (3.44)
China Medical University	4 (3.44)
Grade	
Grade 4	1 (0.86)
Grade 5	99 (85.34)
Postgraduate students	16 (13.79)
Have you taken stomatology courses?	
Yes	115 (99.14)
No	1 (0.86)

### The evaluation of participants’ stomatology English ability based on knowledge quiz session

To evaluate the participants’ English ability, the comprehension of and reaction time to Chinese and English questions in the knowledge session was examined. We selected 96–97 subjects who participated in the knowledge quiz session. The results showed that 82.29% of the participants thought the Chinese questions could be fully understood, while only 7.72% thought the English questions could be fully understood. A total of 63.92% of the participants thought that the response speed for English questions was much slower than that for Chinese questions.

The English ability of undergraduate and postgraduate volunteers was compared based on the knowledge quiz session of the Guanghua Cup. The questions in Table 2 were calculated on a scale of one to five, and the scores of the undergraduate and postgraduate volunteers were compared. The scores for Chinese question understanding (U = 28, *P =* 0.48), English question understanding (U = 35, *P =* 0.96) and reaction time to English and Chinese questions (U = 31.5, *P =* 0.67) between the undergraduate and postgraduate students were not statistically significant.

**Table 2:** Evaluation of participants’ stomatology English ability based on knowledge quiz session

Questions	Number(%)
In the knowledge quiz session, you can completely understand the Chinese questions (answer if you have participated the session)	*n* = 96
Totally disagree	1 (1.04)
Disagree	2 (2.08)
Not sure	2 (2.08)
Agree	12 (12.50)
Totally agree	79 (82.29)
In the knowledge quiz session, you can completely understand the English questions (answer if you have participated the session)	*n* = 97
Totally disagree	5 (5.15)
Disagree	37 (38.14)
Not sure	22 (22.68)
Agree	26 (26.81)
Totally agree	7 (7.22)
In the knowledge quiz session, your reaction to English questions is as fast as your reaction to Chinese questions (answer if you have participated the session)	*n* = 97
Totally agree	0 (0)
Agree	22 (22.68)
Not sure	2 (2.06)
Disagree	62 (63.92)
Totally disagree	11 (11.34)

The comparison of the answer rate (answered questions in proportion of the total English questions) and accuracy (correct answer in proportion of the answered questions) of 8 English questions between the Chinese and international teams in the knowledge quiz session was statistically described. Twenty-two Chinese teams and one international team vied to answer the eight English questions using the buzzers. The Chinese team answered two questions (answer rate: 25%) with one correct answer (accuracy: 50%). The international team answered 6 questions (answer rate: 75%) with four correct answers (accuracy: 66.67%).

### The use of English in stomatology English courses in China

We investigated the status quo of stomatology English education in participating schools from the aspects of the language used by the teachers, textbooks, references and exams. A total of 115 subjects from 22 stomatology schools in China who had already taken stomatology English courses were studied.

The results showed that 63.48% of the participants thought that teachers spoke a little English in the class, 35% of participants used Chinese textbooks, 60.00% of participants used references in mostly Chinese and a little English, and 55.65% of participants took the exam in mostly Chinese and a little English. The statistical descriptions for existing oral English education are shown in Table 3.

**Table 3:** The use of English in a stomatology English course (*n* = 115)

Questions	Number(%)
The teachers in the stomatology English class	
Speak Chinese only	11 (9.57)
Speak a little English	73 (63.48)
Half and half	3 (2.61)
Speak a little Chinese	23 (20.00)
Speak English only	5 (4.35)
The textbooks are	
In Chinese	41 (35.65)
Mostly Chinese and a little English	40 (34.78)
Half and half	3 (2.61)
Mostly English and a little Chinese	10 (8.7)
In English	21 (18.26)
The references are	
In Chinese	2 (1.74)
Mostly Chinese and a little English	69 (60.00)
Half and half	1 (0.87)
Mostly English and a little Chinese	28 (24.35)
In English	15 (13.04)
The exams are	
In Chinese	8 (6.96)
Mostly Chinese and a little English	64 (55.65)
Half and half	5 (4.35)
Mostly English and a little Chinese	11 (9.57)
In English	27 (23.48)

The use of English from each stomatology school was given a score that was defined by the author as the stomatology English education score based on Table 3. Eighty contestants from 22 stomatology schools were included. The score of each stomatology school of the university and the ranking of the Chinese stomatology school based on the 4th China University Subject Rankings (CUSR) by the Center for Degree and Graduate Education Development of the China Ministry of Education in 2017 [11] are shown in Table 4. The correlation of the score and the ranking of the stomatology school in China was examined using the Spearman test. The results show that the relationship between the use of English in the course and the ranking of the stomatology school did not differ significantly (Rs = 0.315, *P =* 0.189).

**Table 4:** Stomatology English education score of each stomatology school (*n* = 80)

University	Number of contestants	Stomatology English education score (from high to low)	CUSR
Sichuan University	2	4.75	2
Tianjin Medical University	4	4.56	12
Guangzhou Medical University	3	4.25	N/A
Guangxi Medical University	4	4.13	17
Chongqing Medical University	4	4.13	18
Tongji University	3	3.75	15
Harbin Medical University	4	3.44	14
Capital Medical University	4	3.13	8
Wuhan University	4	2.94	6
Jilin University	4	2.81	13
Dalian Medical University	4	2.75	16
Xinjiang Medical University	4	2.69	N/A
Shanghai Jiao Tong University	3	2.67	4
Zhejiang University	3	2.33	10
China Medical University	4	2.31	9
Shandong University	3	2.17	11
Nanjing Medical University	4	1.94	5
Peking University	4	1.88	1
Southern Medical University	4	1.88	N/A
Xian Jiaotong University	4	1.81	19
Sun Yat-sen University	3	1.75	7
Air Force Medical University	4	1.63	3

### The influence of stomatology English education on Guanghua cup activity

We investigated how the participants thought about current stomatology English education with regard to helping them communicate and understand in the Guanghua Cup activity. The participants’ ability to understand English was evaluated. A total of 115 subjects who had already taken stomatology English courses were studied.

Regarding how the stomatology English class helped the participants in the Guanghua Cup activity, 45.83% of the students thought that stomatology English education in class was of considerable help in communicating with international students and teachers. In addition, 47.83% of the students thought that it was of considerable help in understanding the Guanghua Cup activity. Vocabulary was found to be the most helpful (98, 85.22%). To understand the detailed aspects of how the stomatology English class helped the participants, the correlation of Table 3 (teachers, textbooks, references and exam) and Table 5 (communication and understanding) was examined using Spearman’s test. The results show that the English textbook score was positively correlated with the score for international communication between teachers and students (Rs = 0.348, *P* = 0.016).

The statistical results demonstrating the influence of the current stomatology English education on the Guanghua Cup activity are shown in Table 5.

**Table 5:** The influence of current stomatology English education on the Guanghua Cup activity

Questions	Number(%)
Did the stomatology English class help you in communication with international students or teachers? (answer if you have communication experience)	*n* = 48
No help	0 (0)
A little help	4 (8.33)
Moderate help	13 (27.08)
Much help	22 (45.83)
The most help	9 (18.75)
Did the stomatology English class help you in understanding English during the Guanghua Cup activity?	*n* = 115
No help	5 (4.35)
A little help	14 (12.17)
Moderate help	27 (23.48)
Much help	55 (47.83)
The most help	14 (12.17)
What do you find the most helpful in the stomatology English class for understanding the Guanghua Cup activity?	*n* = 115
Vocabulary	98 (85.22)
Listening	27 (23.48)
Reading	39 (33.91)
Speaking	15 (13.04)
Writing	5 (4.35)
Other	0 (0)

### The influence of Guanghua cup activity on the participants’ English ability

The influence of Guanghua Cup activities on participants’ English ability was examined among all the participants (*N* = 116, 100.00%). The results showed that 43.97% of the participants had a little English preparation before the Guanghua Cup activity, and 58.62% of the participants said that they had no chance to communicate with international students or teachers. Approximately 30% of the participants said they saw little or no improvement in vocabulary, speaking, listening and reading.

Regarding the participants perception of how they could improve their stomatology English ability, 82.76% of the participants thought that English textbooks and essays helped to improve their stomatology English ability the most, followed by courses focusing on stomatology English (*N* = 81, 69.83%) and participating in an international competition, exchange program, or international conference (*N* = 80, 68.97%). In addition, participants suggested holding an ‘oral stomatology English competition’ and ‘increasing the variety of the forms of stomatology English exams’. The statistical results of the influence of activities on participants’ English ability and perception are shown in Table 6.

**Table 6:** The influence of the Guanghua Cup activity on participants’ English ability (*n* = 116)

Questions	Number(%)
I studied a lot of English for the Guanghua Cup activity before the event	
Totally disagree	19 (16.38)
Disagree	51 (43.97)
Not sure	2 (1.72)
Agree	37 (31.9)
Totally agree	7 (6.03)
I had a lot of communication with the international students and teachers	
Totally disagree	68 (58.62)
Disagree	32 (27.59)
Not sure	1 (0.86)
Agree	11 (9.48)
Totally agree	4 (3.45)
I had great improvement in vocabulary in the Guanghua Cup activity	
Totally disagree	27 (23.28)
Disagree	37 (31.90)
Not sure	17 (14.66)
Agree	27 (23.28)
Totally agree	8 (6.90)
I had great improvement in oral English in the Guanghua Cup activity	
Totally disagree	33 (28.45)
Disagree	38 (32.76)
Not sure	13 (11.21)
Agree	27 (23.28)
Totally agree	5 (4.31)
I had great improvement in listening in the Guanghua Cup activity	
Totally disagree	24 (20.69)
Disagree	33 (28.45)
Not sure	14 (12.07)
Agree	39 (33.62)
Totally agree	6 (5.17)
I had great improvement in reading in the Guanghua Cup activity	
Totally disagree	23 (19.83)
Disagree	38 (32.76)
Not sure	19 (16.38)
Agree	33 (28.45)
Totally agree	3 (2.59)
I had great improvement in my stomatology English ability in the Guanghua Cup activity	
Totally disagree	23 (19.83)
Disagree	39 (33.62)
Not sure	13 (11.21)
Agree	33 (28.45)
Totally agree	8 (6.90)
I paid a lot of attention to stomatology English learning before the Guanghua Cup activity	
Totally disagree	5 (4.31)
Disagree	32 (27.59)
Not sure	54 (46.55)
Agree	14 (12.07)
Totally agree	11 (9.48)
I paid a lot of attention to stomatology English learning after the Guanghua Cup activity	
Totally disagree	1 (0.86)
Disagree	0 (0)
Not sure	6 (5.17)
Agree	32 (27.59)
Totally agree	77 (66.38)
In what ways do you think you can improve your stomatology English ability (multiple choices)	*n* = 116
Traditional stomatology English class	27 (23.28)
Courses focusing on stomatology English	81 (69.83)
Reading English textbooks and assays	96 (82.76)
Attending international competition/exchange program/international conference	80 (68.97)
Other (please list)	6 (5.17)

The participants’ perception of the improvement in stomatology English was determined. The subjects were divided into English volunteers (*N* = 9, 7.76%), Chinese volunteers (*N* = 27, 23.27%) and contestants (*N* = 80, 68.97%). The scores of the three groups differed in English preparation before the Guanghua Cup activity (H = 23.87, *P* < 0.001). The score of English volunteers was higher than that of contestants and Chinese volunteers, and the difference was statistically significant (*P* < 0.001). There was no difference between Chinese volunteers and contestants (*P =* 0.064).

The scores of the three groups differed in communication with international students and teachers (H = 24.63, *P* < 0.001). The score of English volunteers was higher than that of contestants and Chinese volunteers, and the difference was statistically significant (*P* < 0.001). There was no difference between Chinese volunteers and contestants (*P =* 0.058).

The scores of the three groups differed in the improvement of comprehensive English ability (H = 17.86, *P* < 0.001), including vocabulary (H = 16.41, *P* < 0.001), speaking (H = 20.48, *P* < 0.001), listening (H = 13.95, *P =* 0.001) and reading (H = 9.2, *P =* 0.01). The scores of English volunteers were higher than those of Chinese volunteers and contestants, and the difference was statistically significant (*P* < 0.001). There was no difference between Chinese volunteers and competitors (*P* = 0.09).

English volunteers, Chinese volunteers and competitors all attached more importance to oral English after the Guanghua Cup activity than before (U = 1969.5, *P* < 0.01).

## Discussion

In this study, we evaluated the current status of Chinese stomatology English education and the stomatology English ability of the participating students based on the Guanghua Cup activity. The results revealed that the participating students apparently had difficulty in English application and confidence, which was reflected by the low registration rate of English volunteers, low answering rate, long response time for English questions and difficulty in understanding the English questions. Moreover, our study showed that there were large differences in stomatology English teaching nationwide. In addition, we found a positive correlation between English textbooks and peer communication, indicating the importance of English textbooks in improving stomatology English. In the case of English volunteers in this study, their perceived English ability was improved considerably. And all participants realized the importance of English after the competition.

The unsatisfactory English stomatology ability of Chinese participants might result from the limited English exposure in stomatology English education. Only a few stomatology schools emphasize the use of English in class. Surprisingly, the use of English in class is not statistically correlated with the reputation of the stomatology school. The Guanghua School of Stomatology, Sun Yat-sen University, has two schooling systems. The five-plus-three schooling system (the original seven-year program) uses Chinese textbooks, English references and English exams. Only a few teachers speak English during the course. For the students in the five-year schooling system, the stomatology courses are mostly in Chinese. The contestants from the Guanghua School of Stomatology were all from the five-year schooling system, which might account for the low stomatology English education score in Table 4. Therefore, Chinese stomatology students cannot receive a standardized stomatology English course with clear objectives and plans since stomatology English education differs not only between schools but also within schools in China. In other ESL (English as a second language) countries such as Japan, stomatology English courses have lacked consensus on the content and teaching methods [12]. This might be a common phenomenon in non-English speaking countries.

Study motivation could influence on the students attitudes towards the subject. Studies revealed that pharmacy students were more motivated during clinical clerkship [13]. Kusurker et al. [14] reported that deep learning was strongly associated with the interest-motivated profile. In our study, the Guanghua Cup activity increased all participants’ attention to stomatology English. The experience of applying stomatology English during the competition should enhance their learning motivation profile in stomatology English. This finding might shed light on how international activities like Guanghua Cup activity can provide study motivation and act as a good extension and supplement to traditional classroom learning. Moreover, the English volunteers in the Guanghua Cup perceived considerable improvement in English. Preparation before a competition such as the Guanghua Cup activity can act as a PBL (problem-based learning) approach for the participating students. PBL is meant to equip students with an integrated set of knowledge, skills, and attitudes [15]. For English volunteers, our school arranged scenario-oriented language training before the competition. Moreover, the Guanghua Cup activity provides practical application scenarios and communication opportunities for English volunteers. Therefore, among all participating students, the English volunteers perceived the most improvement in language skills.

Based on the current findings about the stomatology English education in China, several suggestions can be made. First, the stomatological education section of the Chinese Stomatological Association should determine standards of stomatology English for Chinese stomatology students. These standards should include an introduction to the subjects, the purpose and requirements of the subject and the major methods of assessment. Second, the teaching mode should be broadened, and multiple teaching methods should be applied. More active learning modes, such as peer teaching and learning activities [16], should be considered. Third, students should be encouraged to attend international competitions such as Guanghua Cup activities, or other activities which requires English application.

There were some limitations to this study. It should be noted that the study only analyzed the top stomatology schools in China, so it might not represent the language skill of every stomatology school in China. The conclusions of this study could be limited because the questionnaire designed for this study was in the developmental stage. And the number of participants in the event was limited. Subsequent studies should include students of the stomatology schools in various universities to achieve a more accurate geographical representation.

## Conclusions

The current stomatology English education is insufficient to meet the demands of Chinese students in stomatology English application. International competitions such as the Guanghua Cup activity can increase participants’ enthusiasm for stomatology English learning and provide a platform for English application. Chinese stomatology schools need to strengthen and reach a consensus on stomatology English education. In the future, the reform of stomatology English teaching should consider multiple scenarios to provide students with adequate language learning and application environments.

## Data Availability

The datasets used and/or analysed during the current study are available from the corresponding author on reasonable request.

## Abbreviations

CUSR: China University subject rankings
ESL: English as a second language
PBL: Problem-based learning

## References

[1] Oka H (2018). Perceptions of dental students in Japanese national universities about studying abroad. Eur J Dent Educ.

[2] Jin H (2020). Evaluation of the current situation of English courses in eight-year M.D. program and prospect of reform — taking Sichuan University as an example. Chin J Med Educ Res.

[3] Takenouchi A (2017). Interest in international programmes - a survey of Japanese dental hygiene students and educators. Int J Dent Hyg.

[4] Takehara S (2016). Characteristics of undergraduate dental students in Japan: English competency and willingness to study abroad. Int Dent J.

[5] Rodis OM (2014). A proposed core curriculum for dental English education in Japan. BMC Med Educ.

[6] National Immigration administration. The number of exit and entry documents issued and the number of people entering and leaving The country in the first half of 2018 saw steady year-on-year growth .2018. https://www.nia.gov.cn/n741435/n741517/c759593/content.html, Accessed 27 Sep 2020.

[7] Wang S, Wang H (2011). On the state of college English teaching in China and its future development. Chin Foreign Lang.

[8] Sun J, Zheng JW (2016). Current status of dental English education in China. Shanghai Kou Qiang Yi Xue.

[9] Wu J (2017). Current status and discussion on stomatology specialized English education in Zunyi Medical University. Chin J Conservative Dent.

[10] Zhao K (2010). Quality requirements and quality assurance of english education for 7-year program postgraduates. Zhonghua Kou Qiang Yi Xue Za Zhi.

[11] China Academic Degrees and Graduate Education Information. China University Subject Rankings. 2017. http://www.chinadegrees.cn/xwyyjsjyxx/xkpgjg/2016phden/index.shtml. Accessed 27 Apr 2020.

[12] Rodis OM (2013). Undergraduate dental English education in Japanese dental schools. J Dent Educ.

[13] Yamamura S, Takehira R (2018). An analysis of the relationship between the learning process and learning motivation profiles of Japanese pharmacy students using structural equation modeling. Pharmacy (Basel).

[14] Kusurkar RA (2013). Motivational profiles of medical students: association with study effort, academic performance and exhaustion. BMC Med Educ.

[15] Hung W (2019). A review to identify key perspectives in PBL meta-analyses and reviews: trends, gaps and future research directions. Adv Health Sci Educ Theory Pract.

[16] Rodis OMM, Locsin RC (2019). The implementation of the Japanese dental English core curriculum: active learning based on peer-teaching and learning activities. BMC Med Educ.

